# A Novel Technique to Detect False Data Injection Attacks on Phasor Measurement Units

**DOI:** 10.3390/s21175791

**Published:** 2021-08-28

**Authors:** Saleh Almasabi, Turki Alsuwian, Ehtasham Javed, Muhammad Irfan, Mohammed Jalalah, Belqasem Aljafari, Farid A. Harraz

**Affiliations:** 1 Electrical Engineering Department, College of Engineering, Najran University, Najran 11001, Saudi Arabia; tmalsuwian@nu.edu.sa (T.A.); miditta@nu.edu.sa (M.I.); msjalalah@nu.edu.sa (M.J.); bhaljafari@nu.edu.sa (B.A.); 2 Neuroscience Center, Helsinki Institute for Life Sciences, University of Helsinki, 00014 Helsinki, Finland; ehtasham.javed@helsinki.fi; 3 Promising Centre for Sensors and Electronic Devices (PCSED), Advanced Materials and Nano-Research Centre, Najran University, P.O. Box 1988, Najran 11001, Saudi Arabia; faharraz@nu.edu.sa; 4 Nanomaterials and Nanotechnology Department, Central Metallurgical Research and Development Institute (CMRDI), P.O. Box 87 Helwan, Cairo 11421, Egypt

**Keywords:** cyber-physical security, false data injection attacks, state estimation, phase lock value, phasor measurement units, smart grids

## Abstract

The power industry is in the process of grid modernization with the introduction of phasor measurement units (PMUs), advanced metering infrastructure (AMI), and other technologies. Although these technologies enable more reliable and efficient operation, the risk of cyber threats has increased, as evidenced by the recent blackouts in Ukraine and New York. One of these threats is false data injection attacks (FDIAs). Most of the FDIA literature focuses on the vulnerability of DC estimators and AC estimators to such attacks. This paper investigates FDIAs for PMU-based state estimation, where the PMUs are comparable. Several states can be manipulated by compromising one PMU through the channels of that PMU. A Phase Locking Value (PLV) technique was developed to detect FDIAs. The proposed approach is tested on the IEEE 14-bus and the IEEE 30-bus test systems under different scenarios using a Monte Carlo simulation where the PLV demonstrated an efficient performance.

## 1. Introduction

In recent years, numerous cyber-attacks were launched against electric power systems, which caused power outages, such as the Ukraine blackout on 23 December 2015 and Manhattan, New York blackout on 13 July 2019 [1,2]. Cyber-attacks are aimed to either damage the power grid or to manipulate the grid markets to gain a financial advantage. Such attacks can lead to many wrong decisions to be taken by the control engineers of the electric power grid. Therefore, it is important to investigate, study and analyze such attacks and data manipulation through the techniques of state estimation (SE) to identify those data that has been attacked and manipulated.

The SE is an essential part of the Supervisory Control and Data Acquisition (SCADA) system, where the SCADA uses state estimators to find the actual states of the power grid. These state estimates are then, utilized by the energy management system (EMS) to perform different system operations, such as contingency analysis and optimal power flow.

Traditionally, state estimators obtain grid measurements from remote terminal units (RTUs), which measure the voltage magnitudes, power injections, and power flows. These measurements are used by the state estimator to obtain the voltage magnitudes and angles for the buses in the grid [3].

**Figure 1:**
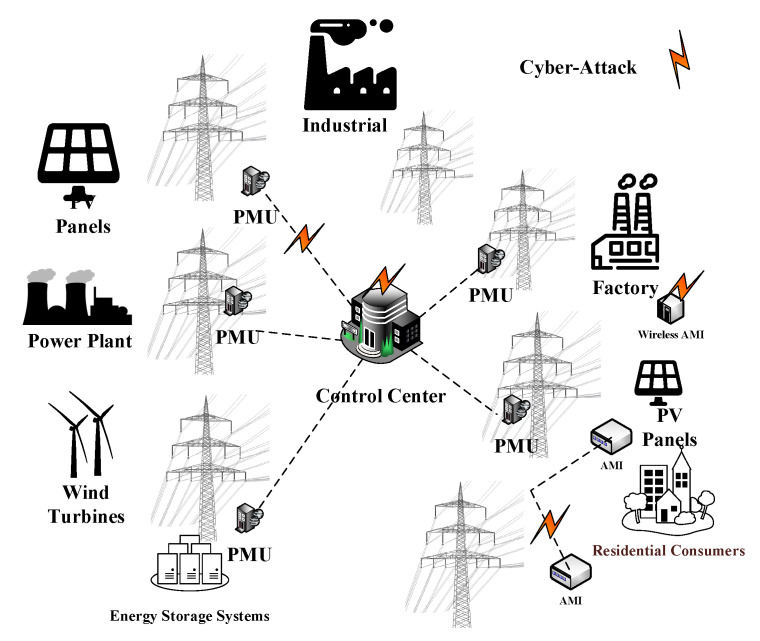
Cyber threats in a smart grid.

The recent advancements of smart meters, such as phasor measurement units (PMUs) and advanced meter infrastructure (AMI), have enhanced the situational awareness and enabled a more secure grid operation. However, these new technologies introduced new vulnerabilities and raised the risk of cyber-threats, as shown in Figure 1. One of these risks is data manipulation, where the adversaries manipulate the measured data to change the system operating conditions, thereby, damaging the grid operations. Such cyber-attacks are known as false data injection attacks (FDIAs).

The FDIA poses are a real threat due to its ability to bypass bad data detection (BDD), thereby changing the system operations without being detected [4]. The BDD is used to detect outliers by using residual-based methods [3]. However, FDIAs utilize the power grid topology to mask the false data and bypass BDD [5].

Most of the FDIA literature focuses on RTU measurements and the DC estimator framework. Teixeira et al. [6] used random FDIAs to evaluate the performance of the BDD in state estimators. Protecting a minimum subset of measurements to guard against FDIAs was proposed by [7,8]. Wang et al. [9] proposed a systematic topology switch of the network for detecting FDIAs.

FDIAs can also be used on AC-estimators, although it is harder to bypass the BDD due to the nonlinearity of these estimators [10,11]. Masking FDIAs with Line outages was investigated in [12], where the adversaries require limited knowledge of the grid topology. Based on the signal processing technique, wavelet singular entropy (WSE) is employed for the detection of any false data injection in the AC systems [13]. Wireless sensor networks (WSNs), including cyber-physical systems (CPSs), were implemented for detection of the distributed attacks of false data injection and jamming attacks [14]. Theoretical analysis for an imperfect FDIA model based on a forecasting-aided method was introduced in [15]. The above-mentioned references are considered RTU-based FDIA in an AC system setting.

Over the last decade, the PMUs started emerging as a better option for grid monitoring over the legacy RTUs, due to their precise measurements, ability to measure phasors and high refresh rate [16]. As a result, several researchers have investigated PMUs vulnerability to FDIAs. The ability to spoof the global positioning system (GPS) signal of PMUs was assessed by [17,18] where several techniques were introduced. The Low Rank Matrix (LRM) factorization method was introduced by [19], to identify false data injection attacks on PMUs. It is shown that the proposed method was able to identify proper power system operation states as well as detect the malicious attacks.

However, later research on LRM [20] demonstrated that a more sophisticated attacker that understands the temporal correlation of PMU data can exploit it to design unobservable FDIA attacks that cannot be detected by the LRM detector. The authors of [21] proposed an optimal placement approach where, by securing a minimum number of PMUs, FDIAs are infeasible. Ding et al. [22] developed a probabilistic model for cyber-threats on PMUs and used an optimal PMU placement to enhance the observability under such a threat.

The optimal placement of PMUs (OPP) using an integrated linear programming (ILP) algorithm to prevent the FDIAs was presented by [23]. It was discovered that a weak power grid can be transformed into a robust power grid by adding a few PMUs at vulnerable locations.

By looking at the literature of FDIAs, most studies are considering RTU-based FDIAs. These studies were performed in either a DC estimator or AC estimator setting. The PMUs were typically used as redundant units to secure the RTU measurements against FDIAs [21]. PMUs have also been used as a source of online data to forecast and develop FDIAs detection techniques under an RTU-based estimator [24]. In [25], a detector for FDIA attacks on hybrid estimators contingent upon the absence of outliers in PMU data.

As discussed earlier PMUs were considered as a backup or a secure platform against FDIA, and the impact of compromising PMUs has not been considered before. In this paper, the effect of attacking state estimators via PMUs data is considered. The strategy for attacking via PMUs and its impact on the state estimators is investigated.

The paper also proposes a detection mechanism for the FDIAs based on a synchronization metric named the phase lock value (PLV) [26]. The PLV was originally proposed in the field of neuroscience to investigate the signals from two or more distinct brain regions whether they are functionally connected or not [27,28], The PLV quantifies the synchronicity present between two signals based on phase changes [29,30,31], where the underlying assumption is that, for a certain time-period, if the phase changes of two signals are consistent, they are said to be connected/synchronized, and PLV will result in a value closer or equal to ‘one’.

Whereas, if phase changes do not show consistency, two signals are not connected and for such PLV will have a value closer or equal to ‘zero’. With this background in mind, PLV can be utilized to study unwanted randomness between signals/data. For example, consider two connected signals resulting in a consistent phase change, but when randomness is added to one of the signals, then the differences in phases are no longer constant, and thus two signals are no longer connected to each other. On similar lines, Patrick Celka [31] showed that different types of noise processes affect PLV differently and the strength of noise enhances between-processes effects. However, common among noise processes, it could be noticed that, with the introduction of noise, the PLV tends toward zero implying that the underlying signals deviate from being synchronized.

This motivated us to utilize this concept in the identification of FDIA, and we hypothesized that under normal circumstances, when there is no data manipulation, the buses in the grid will have consistent phase changes between them, whereas, in the case of manipulated data, the differences between phases will no longer be constant. The proposed approach is tested on the IEEE 14-bus and the IEEE 30-bus test systems under different conditions using Monte Carlo simulation.

The main contributions of this article can be summarized as follows:

- Most of the existing FDIAs assume DC model associated with RTUs. In RTU-based attacks, the adversaries need to compromise several RTUs, where PMU-based attacks compromising one PMU are sufficient for a successful attack. This paper addresses PMU-based FDIAs.
- This presents an effective approach for detecting FDIA attacks using PLV.
- The proposed approach requires no training to build a model, and can be used online to detect FDIAs.

The rest of the paper is organized as follows. Section 2 describes state estimation in the presence of PMUs. Section 3 discusses the attack strategy for FDIA. Section 4 presents the proposed PLV detection mechanism. Section 5 presents the simulation results, and Section 6 concludes the paper.

## 2. State Estimation

State estimators use the measurements obtained for the RTUs or the PMUs to find the voltage magnitudes and angles for the buses ($\hat{x}$). If the grid is completely observable by the PMUs, the state estimation becomes a linear process [3,32]. For the process to be linear, the state and measurements vectors ($\hat{x}$, *z*) in (1) are considered to be in the rectangular form (real and imaginary). State estimators use the data from either RTUs or PMUs, then, based on the acquired data, the state estimation process becomes linear or nonlinear. The RTUs measure the voltage magnitudes, power flows, and power injections. The PMUs on the other hand, measure the voltages of the buses and current flows in phasor form. The measurement model can be described as follows

(1) $$z_{p}\left(t\right)=H\hat{x}\left(t\right)+v\left(t\right),$$

where $z_{p}\left(t\right)$ is the measurement vector at time *t*; the *t* is dropped for convenience. H is the transition matrix, $\hat{x}\left(t\right)$ is the state vector, and *v* is the measurement noise [3].

In PMU-based state estimation, the measurement vector $z_{p}$ is arranged in a rectangular form to enable a linear estimation process, $z_{p}={\left[V_{i}^{real}V_{i}^{imag}.....\;I_{m}^{real}I_{m}^{imag}\right]}^{T}$[3,32]. The same arrangement is applied to the state vector $\hat{x}$ as follows

(2) $$\hat{x}={\left[V_{1}^{real},V_{1}^{imag}......V_{n}^{real},V_{n}^{imag}\right]}^{T}.$$

By using this arrangement the transition matrix H becomes an *m* by 2n constant matrix with two parts, where *m* and *n* are the number of measurements and buses, respectively. The first part is the identity matrix I corresponding to the direct measurements of bus voltages by the PMUs. The second part is a sub-matrix corresponding to the current measurements as in (3).

(3) $$\left[\begin{array}{c} I_{m_{v}\times 2n}\\ H_{\alpha_{m_{i}},\beta_{m_{i}}\times 2n} \end{array}\right].$$

where, $m_{v}$ and $m_{i}$ are the number of voltage and current measurements respectively. $h_{\alpha }$ and $h_{\beta }$ are the matrices of the branch admittance $Y_{ij}$ decomposed such that $h_{\alpha }$ produces the real part of the branch current $I_{ij}$, and $h_{\beta }$ produces the imaginary part of the branch current $I_{ij}$. Therefore, $h_{\alpha }$ and $h_{\beta }$ for the current $I_{ij}$ can be expressed as follows

(4) $$h_{\alpha_{ij}}=\left[0\ldots G_{ij}\;-\;G_{ij}\;0\cdots -\;B_{ij}\;\;-\;B_{ii}\;\;B_{ij}\;0\ldots \right];$$

(5) $$h_{\beta_{ij}}=\left[0\cdots -\;\;B_{ij}\;\;+\;B_{ii}\;\;B_{ij}\;0\ldots G_{ij}\;\;G_{ij}\;0\ldots \right];$$

By using the model described above the states $\hat{x}$ can be determined using weighted least squares as follows:

(6) $$\hat{x}={\left(H^{T}R^{-1}H\right)}^{-1}H^{T}R^{-1}z_{p};$$

where *R* is the covariance matrix of the noise.

## 3. Attack Model

This section describes FDIAs for RTU-based and PMU-based state estimators. Different notations will be used for both estimators, as they defer in terms of the type of measurements and transition matrices. For the RTU-based attacks, $z_{R}$ and *H* will be used to refer to the measurement and transition matrix. As for the PMU-based attacks, $z_{p}$ and H will be used to refer to the measurement and transition matrix.

### 3.1. RTU-Based Attack Models

In DC-estimators, the voltage magnitude of all the buses in the grid is assumed to be equal to one p.u., and the angle difference between the buses is assumed to be less than five degrees. Therefore, the measurement model for DC-estimators becomes

(7) $$z_{R}=Hx_{DC}+v;$$

where $z_{R}$ and *v* are the measurement and noise vectors, respectively, with a size of *m* by one. In DC-estimators, $z_{R}$ is an *n* by one vector whose elements are the power flow and power injection described in (8). $x_{DC}$ is the vector of bus angles θ with a size equals to the number of buses *n*. *H* is constructed to correspond to the following model:

(8) $$\begin{array}{c} P_{ij}=\frac{\theta_{i}-\theta_{j}}{\mathrm{Reactance}\;\mathrm{of}\;\mathrm{line}\;ij}\;;\\ P_{i}=\sum P_{ij}. \end{array}$$

Under the DC-estimators paradigm, the adversaries try to manipulate the measurement vector $z_{R}$ in (7) while avoiding detection by the BDD in (9). This manipulation should be less than the tolerance (τ) of the residual to avoid detection. Therefore, the sparse attack vector (*a*) in (10) should be a=c×h, where h∈H and *c* is the desired manipulation by the adversaries. By using *a* the residual for the BDD remains the same as shown in (11).

(9) $$∥z_{R}-Hx_{DC}∥\leq \tau$$

(10) $$z_{R,comp.}=z_{R}+a$$

As seen in (11), by using such a vector the regular BDD can no longer detect the FDIA [7,8,9]. However, the adversaries need to have partial knowledge of the grid topology to use such a vector.

(11) $$\begin{array}{c} ∥z_{R,comp.}-Hx_{comp.}∥=∥z_{R}+a-H\left(x_{DC}+c\right)∥\\ =∥z_{R}+H\times c-H\times x_{DC}-H\times c)∥\\ =∥z_{R}-H\times x_{DC})∥\leq \tau . \end{array}$$

In AC-estimators, the voltage magnitudes are no longer assumed as in the DC-estimator but estimated. The measurement vector *z* consists of voltage magnitudes, power flows, and power injections. These measurements make the state estimation a nonlinear process since (*H*) becomes a nonlinear function of the states (*x*) as in (12). Solving for the states *x* is done iteratively, in a similar process to that of the power flow by using the Jacobian matrix *J* and updating both the vector of the states *x* and *J*.

(12) z=H(x)+v.

The AC-estimators uses the normalized residual for BDD in (9). However, since the states are not linearly dependent on *H*, the attack vector (*a*) needs to be a function of *H* to avoid detection. The FDIA can be implemented by making *a* as follows

(13) $$z_{comp.}=z_{true}+a;$$

where

$a=h(x_{comp.})+H(x_{true}),$

_*true*_ subscript indicates true (uncompromised) state or measurement,

_*comp.*_ subscript indicates compromised state or measurement.

As a result, the attack vector compromises the states without being detected as in (14) [11].

(14) $$\begin{array}{c} ∥z_{comp.}-H\left(x_{comp.}\right)∥=∥z_{true}+a-H\left(x_{comp}\right)∥=\\ ∥z_{true}+H\left(x_{comp.}\right)-H\left(x_{true}\right)-H\left(x_{comp.}\right)-2H\left(x_{true}\right))∥\\ =∥z_{true}-Hx)∥\leq \tau . \end{array}$$

### 3.2. PMU-Based Attack Model

The previous section describes FDIAs for RTUs where several units need to be manipulated for a successful attack. PMUs, on the other hand, have several channels where a single PMU can measure the bus voltage and all adjacent bus currents in phasor form. This feature enables linear state estimation. However, in the context of FDIAs compromising one PMU is sufficient for launching successful attacks. As for RTU-based attacks, the adversaries need to compromise/manipulate several RTUs. The measurement model for the PMUs can be described as follows:

(15) $$z_{p}=Hx+v.$$

To launch such attacks, the measurements vector $z_{p}$ in (15), which consists of the bus voltages and current flows can be changed using the same approach described in Section 3.1. By using the grid topology, the attack vector can be masked, thereby, bypassing the BDD. The grid topology (H) can be estimated by monitoring the measurements of the targeted PMUs, and there is no need for estimating the whole grid topology. Only local topology (h∈H), is needed for launching successful FDIAs. The attack vector can be constructed as follows

(16) $$z_{comp.}=z_{true}+a;$$

where

$a=c\times {\left[0...h_{1}h_{2}...h_{i}0...0\right]}^{T},$

$z_{comp.}={\left[z_{{true}_{1}}z_{{true}_{2}}...z_{{comp}_{1}}z_{{comp}_{2}}z_{{comp}_{i}}z_{{true}_{i+1}}\right]}^{T},$

*h*_i_ is a subset of H.

By using this vector the residual in (17) remains unchanged.

(17) $$\begin{array}{c} ∥z_{comp.}-Hx_{comp.}∥=∥z_{true}+a-H\left(x+c\right)∥\\ =∥z_{true}+\mathrm{hc}-Hx-\mathrm{hc}∥=∥z_{true}-hx)∥\leq \tau . \end{array}$$

Therefore, as long as the adversaries adhere to the vector in (16), the FDIA will be successful. One common factor between RTU-based and PMU-based attacks is the reliance on the network topology. This information can be obtained through disgruntled employees or through monitoring the data stream. The differences between RTU-based and PMU-based attacks are as follows: 1. RTUs are easier to compromise; however, the adversaries need to compromise several RTUs depending on the network topology. As for the PMUs, they are harder to compromise but compromising one PMU is sufficient. 2. In RTU-based attacks, the aim is to change the bus angles, as the voltage magnitudes are assumed to be constant. In the PMU-based attack, on the other hand, both the voltage magnitudes and angles can be targeted.

## 4. Detection of FDIAs

This section presents the PLV approach for detecting FDIAs. Numerous studies in the field of neuroscience have studied synchronization between two signals from distinct brain regions, and the commonly used measure is PLV [26]. It measures the phase interaction between complex signals using the following:

(18) $$PLV\left(t\right)=|E\left(e^{j\varphi_{12}\left(t\right)}\right)|$$

where φ(t) is the phase difference $\varphi_{12}\left(t\right)\;=\;\theta_{1}\left(t\right)-\theta_{2}\left(t\right)$, E[.] denotes the expected value, and the PLV is estimated at time t. The phase $\theta_{1}$ and $\theta_{2}$ are the phases of the following signals:

(19) $$\begin{array}{cc} x_{1}\left(t\right) & =A_{1}\left(t\right)e^{j\theta_{1}\left(t\right)}\\ x_{2}\left(t\right) & =A_{2}\left(t\right)e^{j\theta_{2}\left(t\right)}. \end{array}$$

**Figure 2:**
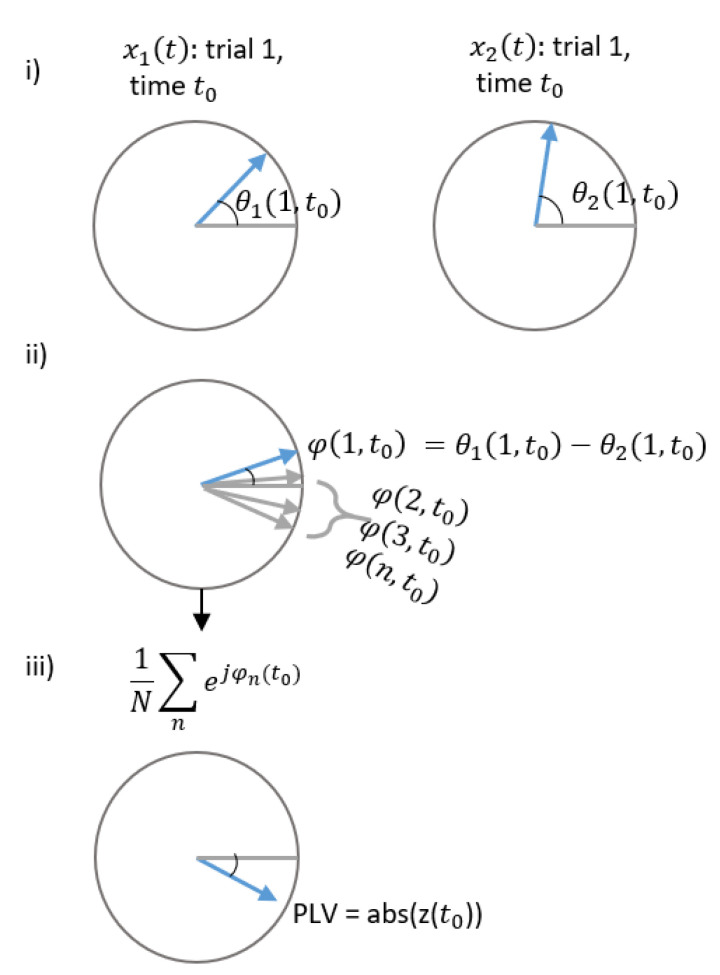
Correlated signals and corresponding PLV. (i) phases of a single trial of two complex-value signals at $t_{o}$, (ii) difference between phases for multiple trials is presented (iii) The resulting in complex PLV for the correlated signals.

**Figure 3:**
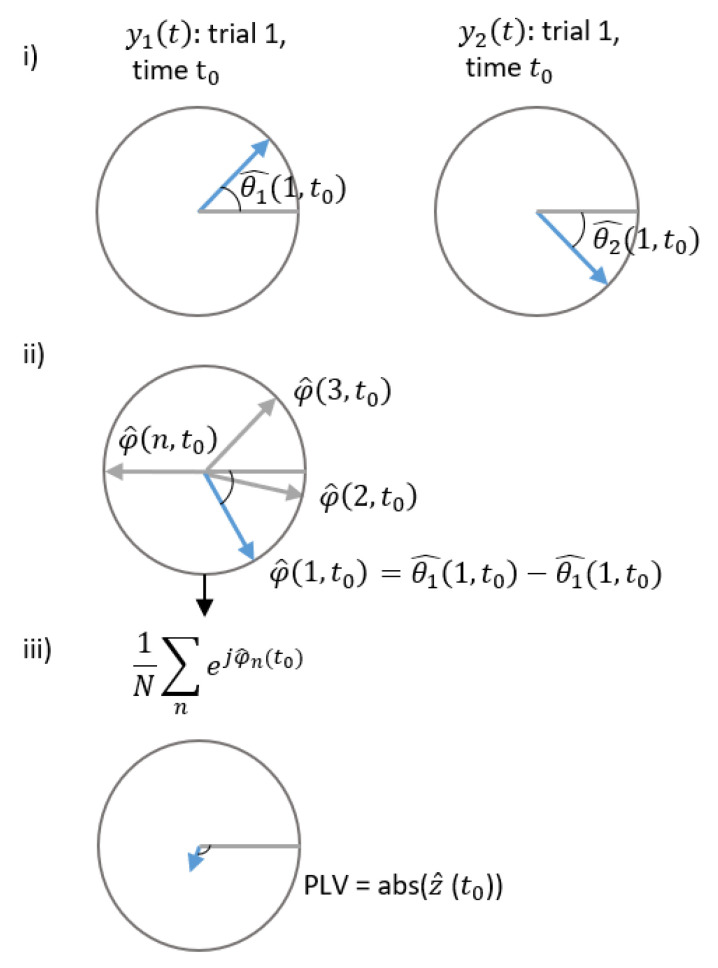
Uncorrelated signals and corresponding PLV. (i) phases of a single trial of two complex-value signals at $t_{o}$, (ii) difference between phases for multiple trials is presented (iii) The resulting in complex PLV for the uncorrelated signals.

The PLV ranges [01] where 0 represents huge variability between phases or in other words no synchrony, and 1 describes identical phases, i.e., synchrony. See Figure 2 and Figure 3 for a visual description. Figure 2 is an example of correlated signals and corresponding PLV, where: (i) phases of a single trial of two complex-value signals at $t_{0}$, (ii) difference between phases for multiple trials is presented, and (iii) resulting in complex PLV, whereas its magnitude, abs(), gives the resulting PLV. The same is repeated in Figure 3 to show the resulting small PLV for uncorrelated signals. In this article, Equation (18) is utilized to develop an analytical detection procedure of FDIA. Algorithm 1 describes the steps involved.

**Algorithm 1:** PLV-based FDIA detection

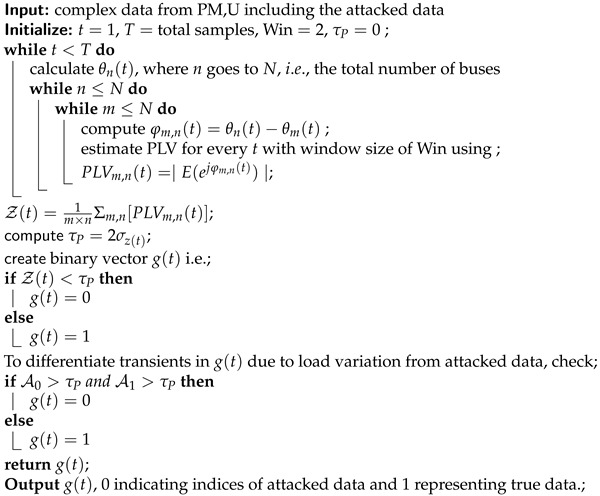



The proposed algorithm makes certain assumptions for the detection of FDIA. It includes: (i) at least the first three time-samples of input data are not attacked, (ii) since the PLV is calculated at each time-sample with a window of size ‘2 time-samples’, the attacked segments should be separated by a segment of three true data samples. Otherwise, if there are one or two true data samples between two attacked segments, the proposed method will consider them as attacked also.

**Figure 4:**
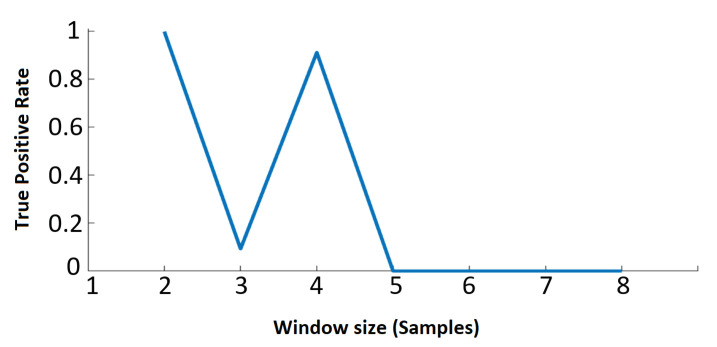
True positive rate by varying window sizes for the calculation of PLV.

Here, it is also important to highlight that we tested different window sizes for the PLV calculation, and the best results were found for the window size = 2 samples as shown in Figure 4. We used the ‘True Positive rate’ to show how variable window sizes affect the predicted outcome. These assumptions are not substantial compared to the requirements in existing studies, such as a large amount of non-attacked historical data to train classifiers [8,9].

**Figure 5:**
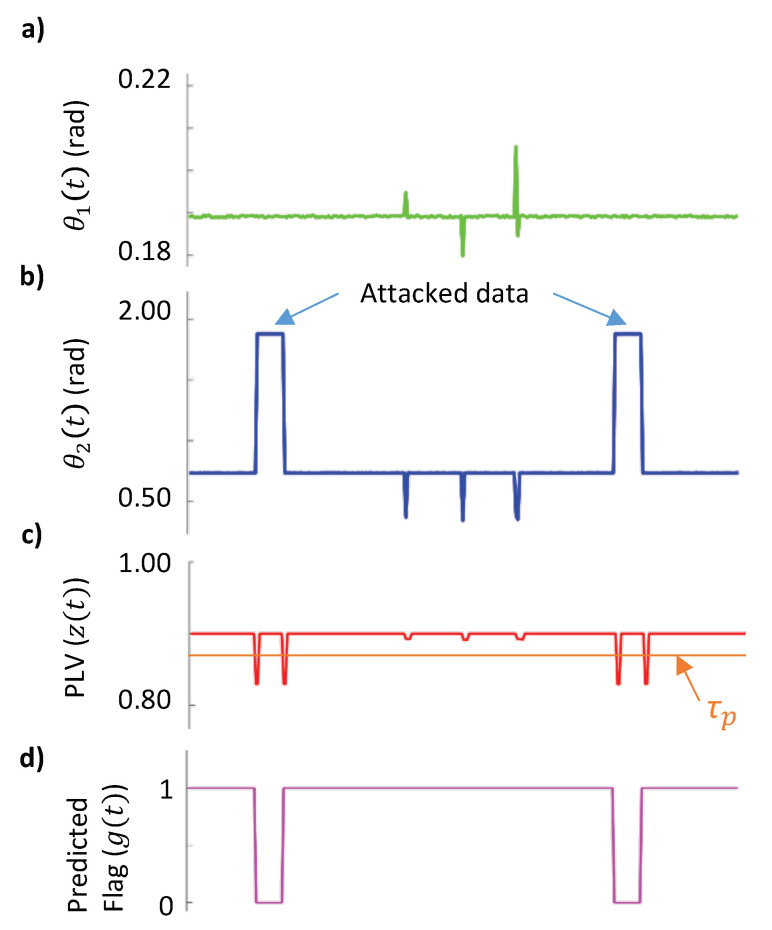

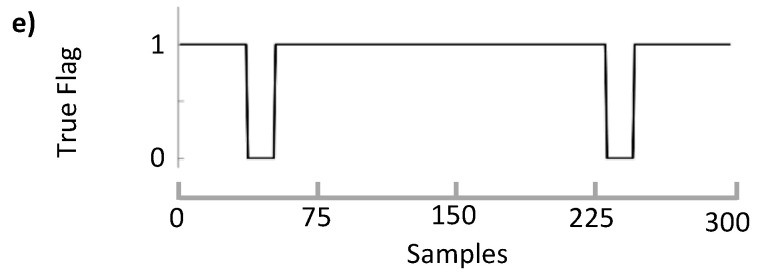
Example of false data detection using PLV. (**a**,**b**) are instantaneous phases $\theta_{1}\left(t\right)$, $\theta_{2}\left(t\right)$ over a time-length of 300 samples. (**c**) Phase lock value between two signals, spikes appear when there is a change in phases of a given signal. (**d**) Based on the proposed method, false data injected in (**b**) are predicted (Flag value ‘0’ highlights attacked samples). (**e**) A waveform representing the ground truth is shown as a reference.

Figure 5 shows an example of false data detection using the proposed method over a simulated data of two buses from above mention the network topology: (a) instantaneous phases $\theta_{1}\left(t\right)$ for the first signal having sudden changes due to load variations, (b) instantaneous phases $\theta_{2}\left(t\right)$ for the second signal that has attacked samples and changes in phases due to load variation between attacked samples.

This is to show that the proposed method is capable of differentiating between attacked samples and samples with phase changes due to load variations. (c) The absolute values of PLV for each sample between (a) and (b) are shown along with the threshold $\tau_{P}$, which is calculated as 2× the standard deviation present in $z_{p}\left(t\right)$. (d) The predicted flag g(t) i.e., samples that are not attacked and samples that are attacked, estimated using proposed method is provided (e) for ground truth, the Flag with true labeling of samples is presented.

(20) $$g\left(t\right)=\left\{\begin{array}{c} 0,\;\;\;\mathrm{if}\;A_{0}>\tau_{P}\;\;\&A_{1}>\tau_{P}\\ 1,\;\;\;\mathrm{else} \end{array}\right.$$

where $A_{0}=Z\left(t_{i-1}\right)-Z\left(t_{i}\right)$; $A_{1}=Z\left(t_{i+2}\right)-Z\left(t_{i+1}\right)$.

## 5. Simulation and Results

**Figure 6:**
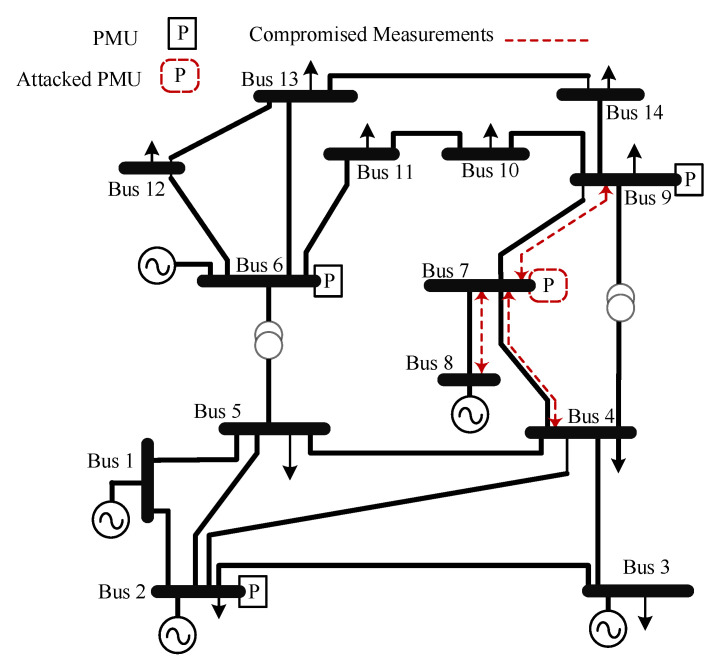
IEEE 14-bus with PMU locations.

**Figure 7:**
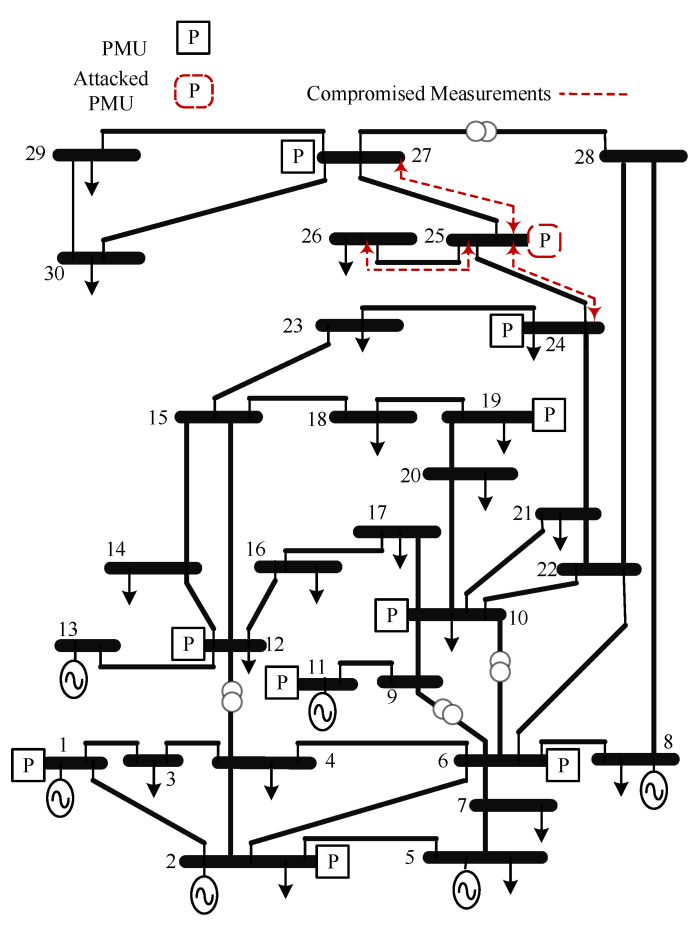
IEEE 30-bus with PMU locations.

This section presents the PLV approach for detecting PMU-based FDIAs. The approach is carried out on the IEEE 14-bus and the IEEE 30-bus test systems. The FDIAs are tested on both systems using the approach mentioned in Section 3.2. The test systems and PMU locations are shown in Figure 6 and Figure 7. The PMU locations were chosen to achieve complete observability under normal conditions [33,34,35], where each PMU measures the currents of all adjacent buses and the voltage of the bus of the PMU. Zero injection buses are not considered in PMU placement.

In the proposed approach, only the current data are processed to detect FDIAs. By ignoring the voltage data, the computation efficacy is enhanced, without affecting the accuracy of the detection. The adversaries need to use the attack vector *a* in (16), otherwise the BDD in (17) will catch this manipulation as outlier data. Therefore, processing the current data is sufficient as no successful attacks can be launched without compromising this data.

Each PMU can generate up to 50 samples per second. In this paper, the PMUs are assumed to be sending the data at a 30 Hz rate, and the state estimation is done every second. This assumption means that the state estimator has a measurement matrix *z* of size *m* by 30 available for evaluation.

### 5.1. Performance Metrics

The efficacy of the PLV approach is evaluated using performance metrics resulting from the confusion matrix. As the confusion matrix demonstrates the efficiency of any given method in predicting classes of test data where the ground truth is also known. The confusion matrix is defined as shown in Table 1.

The derivatives from the confusion matrix, which provides quantitative analysis of goodness of the proposed method, are:

(21) $$Accuracy\left(Acc\right)=\frac{TP+TN}{TP+FN+FP+TN}$$

Acc refers to the term that provides a ratio of correctly predicted samples to total samples.

(22) $$Specificity\left(Spec\right)=\frac{TN}{TN+FP}$$

Spec or true negative rate, provides the ratio of correctly identified negatives.

(23) $$Sensitivity\left(Sen\right)=\frac{TP}{TP+FN}$$

Sen or true positive rate, provides the ratio of correctly identified positives.

(24) $$F_{1}score=\frac{2TP}{2TP+FP+FN}$$

where

*TP* normal samples identified correctly (true positive)

*FP* attacked samples identified incorrectly (false positive)

*TN* attacked samples identified correctly (true negative)

*FN* normal samples identified incorrectly (false negative)

$F_{1}score$ is a harmonic mean of the recall and precision, where the recall is the same as Sen, and the precision is the ratio of the number of true positive samples to the number of true plus false positives.

### 5.2. Case Studies

Each PMU is assumed to measure the voltage of bus where the PMU is located, and the currents of all adjacent buses. Each PMU is sending the measurements at a speed of 30 samples per second. The meter errors of PMU measurements follow the normal distribution with a zero mean and standard deviation of $10^{-3}$. The tests are performed on the IEEE 14-bus and IEEE 30-bus test systems. The load of each test system is varied for all scenarios and all Monte Carlo simulations.

- **Scenario I:** In this scenario, the PMU located at bus 7 is attacked by the adversaries, and fifty Monte Carlo simulations are carried out. The attack vector *a* is kept constant for all fifty cases, however, the instant and duration of the attack are random.
- **Scenario II:** In this scenario, the attacked PMU is random, and fifty Monte Carlo simulations are carried out. The attack vector *a* is kept constant for all fifty cases, however, the instant and duration of the attack are random.
- **Scenario III:** In this scenario, the attack vector *a* changes randomly for each Monte Carlo simulation. The attacked PMU is chosen randomly, and the duration of the attack is random.

The results for the IEEE 14-bus test system are shown in Table 2, where the PLV shows consistent results regardless of the scenario complications. As mentioned earlier, each scenario had a total of fifty Monte Carlo simulations, and the results for each case were evaluated using the metrics in Section 5.1. Therefore, Table 2 shows the mean and the standard deviation for all scenarios based on the Monte Carlo simulations. Table 3 shows a sample of the results for **Scenario III** where different PMUs are attacked at random.

For the IEEE 30-bus test system, **Scenario III:** is used to test the validity of the PLV approach. In addition to the increased number of measurements due to the increased number of buses and number of PMUs as shown in Figure 7, the system presents interesting cases where PMUs are located at radial buses, such as bus 10. Therefore, if this particular PMU is attacked, the adversaries will manipulate two signals, which are non-redundant. However, the proposed approach achieved good results as shown in Table 4 and Table 5.

**Figure 8:**
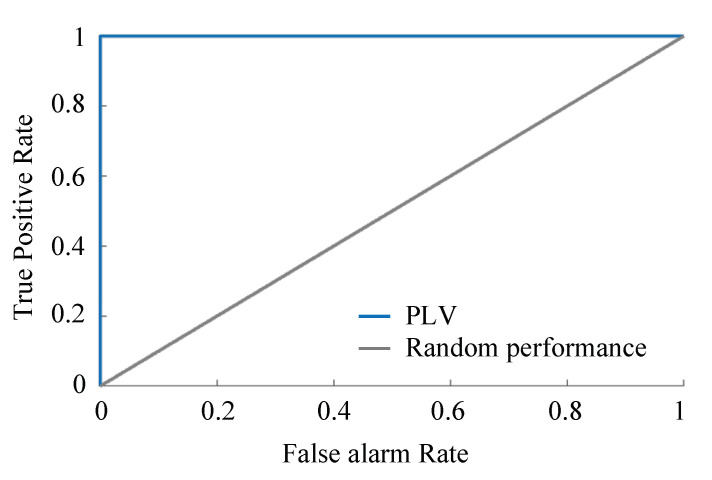
Performance for IEEE-14 system, Scenario III and case number 19 (randomly chosen): ROC curve of the proposed method along with reference ROC curve representing 50% sensitivity and 50% specificity.

**Figure 9:**
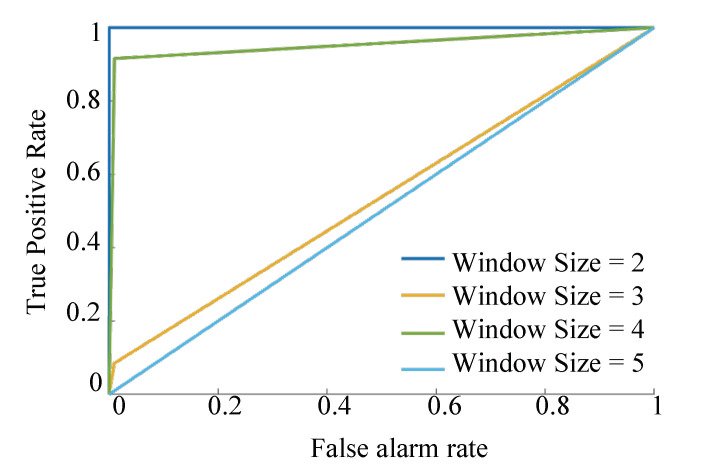
Receiver operating characteristic curve of PLV for different window sizes.

The receiver operating characteristic (ROC) shown in Figure 8, indicates the effectiveness of the PLV as a detection tool for FDIAs. Even in cases where there is a low redundancy the PLV performance is effective—for instance, the case of attacking the PMU of bus 10 where there is one current measurement and one voltage measurement. The window size for the PLV in the above results is two as this is the most effective size. Figure 9 shows the ROC for different window sizes, which indicates that the performance deteriorates as the window size becomes larger. Moreover, the even number window size performances are better than the odd ones. Incidentally, this performance and window size relationship benefits the computation burden as smaller window sizes lead to lesser processing times.

## 6. Conclusions

In this paper, we introduced PMU-based FDIAs where compromising one PMU is sufficient to launch successful attacks and bypass BDD. The paper also introduces a new approach for detecting FDIA where PLV is used to measure the correlation between the measured signals and detect abnormalities. The proposed approach requires no training to build a model and can be used online along with existing BDD. The PLV approach as a detection mechanism was tested on the IEEE 14-bus and IEEE 30-bus test systems using a Monte Carlo simulation with several scenarios where PLV was proven to be an efficient detection tool for FDIAs.

The PLV was used on the current data to decrease the computation burden, and the results demonstrated that using current data was sufficient. In cases where the adversaries change the voltage data without manipulating the current data, the BDD will flag such values as outliers. In the proposed approach, a window size of two was shown to be the best choice as the accuracy of the PLV drops significantly with the larger window sizes.The load change was considered as part of normal operations as such changes are expected during the day. In the PLV approach, the load conditions were varied randomly, and the intensity of the attacks varied to test the robustness of the PLV approach.

As the goal of the adversaries is to change some elements in the state vector by launching FDIAs, which can be done in steady state measurement data The type of measurements and state estimator plays a significant role in launching and detecting FDIAs. One of the future directions is to investigate FDIAs in hybrid estimators where there is a mix of RTU and PMU measurements and the lack of synchronization between RTUs and PMUs adds complexity to the problem.

## Figures and Tables

**Table 1:** Confusion matrix.

	Actual Class	Positive	Negative
Predicted Class			
Positive		True Positive (TP)	False Negative (FN)
Negative		False Positive (FP)	True Negative (TN)

**Table 2:** The mean and standard deviation of the PLV performance for the IEEE-14 bus system.

	Metric	Attack Vector	Attacked PMU	Acc (mean ± std)	Spec (mean ± std)	Sen (mean ± std)	F1-Score (mean ± std)
Case							
**Scenario I:**		constant	7	99.973 ± 0.1155	99.996 ± 0.0165	99.976 ± 0.1155	0.999 ± 0.0090
**Scenario II:**		constant	random	99.992 ± 0.0022	99.992 ± 0.0022	100 ± 0.0000	0.999 ± 0.0011
**Scenario III:**		variable	random	99.972 ± 0.0045	99.973 ± 0.0047	99.999 ± 0.0000	0.998 ± 0.0023

**Table 3:** Scenario III: Sample results for the IEEE-14 bus system.

Case Number	Attacked PMU	Acc %	Spec %	Sen %	F1-Score
7	**7**	99.96806	99.96453	99.67929	0.99839
19	**9**	99.97685	99.97429	99.76812	0.99884
41	**6**	99.98101	99.97894	99.80815	0.99904
2	**2**	99.9686	≈100	99.96864	0.99984

**Table 4:** Scenario III: Sample results for the IEEE-30 bus system.

Case Number	Attacked PMU	Acc %	Spec %	Sen %	F1-Score
1	**1**	99.98333	100	99.98177	0.99991
2	**12**	99.97685	100	99.97453	0.99987
3	**2**	99.98143	100	99.97958	0.99989
4	**8**	99.98380	100	99.98219	0.99991
5	**10**	99.97917	100	99.97705	0.99988
6	**19**	99.97731	100	99.80815	0.99988
7	**24**	99.98148	100	99.97970	0.99989
8	**27**	99.98333	100	99.98163	0.99991
9	**11**	99.98287	100	99.98106	0.99991

**Table 5:** The mean and standard deviation of the PLV performance for the IEEE-30 bus system.

	Metric	Attack Vector	Attacked PMU	Acc (mean ± std)	Spec (mean ± std)	Sen (mean ± std)	F1-Score (mean ± std)
Case							
**Scenario III:**		variable	random	99.9814 ± 0.0029	100 ± 0.000	99.9795 ± 0.0032	0.9897 ± 0.00160

## Data Availability

Not applicable.
